# Toxicity Ranking and Toxic Mode of Action Evaluation of Commonly Used Agricultural Adjuvants on the Basis of Bacterial Gene Expression Profiles

**DOI:** 10.1371/journal.pone.0024139

**Published:** 2011-11-18

**Authors:** Ingrid Nobels, Pieter Spanoghe, Geert Haesaert, Johan Robbens, Ronny Blust

**Affiliations:** 1 Laboratory for Ecophysiology, Biochemistry and Toxicology, Department of Biology, University of Antwerp, Antwerp, Belgium; 2 Department of Crop Protection Chemistry, Ghent University, Ghent, Belgium; 3 Department of Biosciences and Landscape Architecture, University College Ghent, Ghent, Belgium; 4 Department of Plant Production, Ghent University, Ghent, Belgium; Naval Research Laboratory, United States of America

## Abstract

The omnipresent group of pesticide adjuvants are often referred to as “inert” ingredients, a rather misleading term since consumers associate this term with “safe”. The upcoming new EU regulation concerning the introduction of plant protection products on the market (EC1107/2009) includes for the first time the demand for information on the possible negative effects of not only the active ingredients but also the used adjuvants. This new regulation requires basic toxicological information that allows decisions on the use/ban or preference of use of available adjuvants. In this study we obtained toxicological relevant information through a multiple endpoint reporter assay for a broad selection of commonly used adjuvants including several solvents (e.g. isophorone) and non-ionic surfactants (e.g. ethoxylated alcohols). The used assay allows the toxicity screening in a mechanistic way, with direct measurement of specific toxicological responses (e.g. oxidative stress, DNA damage, membrane damage and general cell lesions). The results show that the selected solvents are less toxic than the surfactants, suggesting that solvents may have a preference of use, but further research on more compounds is needed to confirm this observation. The gene expression profiles of the selected surfactants reveal that a phenol (ethoxylated tristyrylphenol) and an organosilicone surfactant (ethoxylated trisiloxane) show little or no inductions at EC_20_ concentrations, making them preferred surfactants for use in different applications. The organosilicone surfactant shows little or no toxicity and good adjuvant properties. However, this study also illustrates possible genotoxicity (induction of the bacterial SOS response) for several surfactants (POEA, AE, tri-EO, EO FA and EO NP) and one solvent (gamma-butyrolactone). Although the number of compounds that were evaluated is rather limited (13), the results show that the used reporter assay is a promising tool to rank commonly used agricultural adjuvants based on toxicity and toxic mode of action data.

## Introduction

Adjuvants are compounds that modify the effects of other compounds without having any direct effects themselves. In most cases they are added to a pesticide formulation to increase the performance of the active ingredients or to make the formulation chemically more stable [1]. Depending on the usage, two different types of adjuvants are distinguished, spray adjuvants and formulation additives. Spray adjuvants also called tank mix adjuvants are added in the spray tank along with the pesticide(s) just before application on the field. The second type of adjuvants called formulation additives or inert ingredients are part of the pesticide formulation [1], [2].

**Figure 1:**
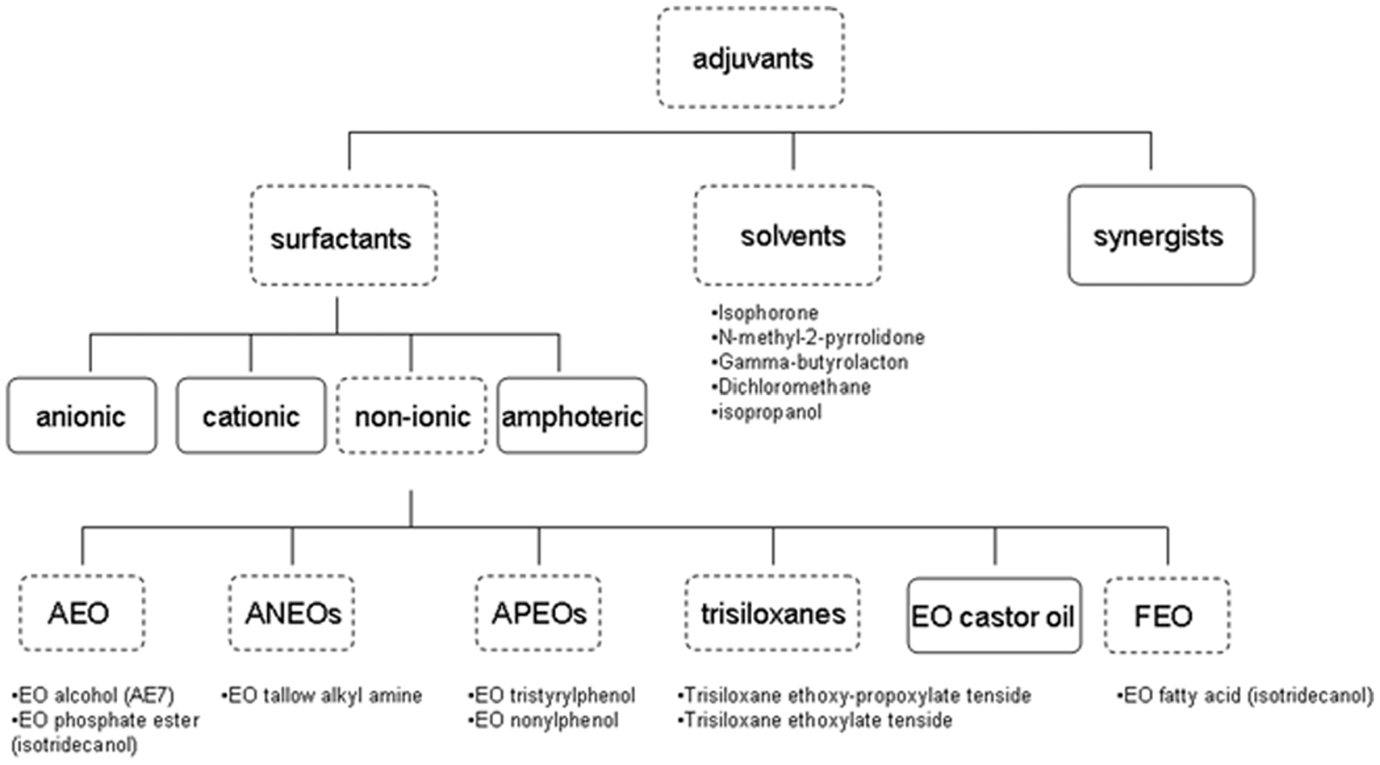
Overview of major types of adjuvants. Dotted squares represent selected groups, below one or more evaluated representatives. APEOs: alkyl phenol ethoxylates, ANEOs: alkyl amine ethoxylates, AEO: alcohol ethoxylates, FEO: fatty acid ethoxylates and EO: ethoxylated.

Besides solvents, surfactants and especially non-ionic surfactants make up the largest group of adjuvants, a simplified overview of the most important chemical classes is listed in Figure 1. This large and heterogeneous group of chemicals is used in pesticides, detergents, personal care and many other products. Due to their variety in applications, adjuvants are the chemicals that are produced and consumed in the largest volumes in the world and most of them end up in detectable levels dispersed in different environmental compartments (soil, water, sediment) and in our food chain [3], [4].

Nevertheless, there is a lack in current (pesticide) legislation concerning the use and allowable residue levels of adjuvants. Current regulation concerning the placing of plant protection products on the market, Directive 91/414/EEC, does not specifically deal with adjuvants. The upcoming new regulation (EG) 1107/2009 replaces the Directives 79/117/EEG and 91/414/EEG and will apply from June 2011. The new regulation acknowledges the need for more (eco)toxicological information regarding all the components of plant protection products and claims a better protection of human, animal and environmental health by applying the precautionary principle. Adjuvants will make part of future pesticide risk evaluations and a list of forbidden adjuvants for use in crop protection will be constructed when more information becomes available. Industry has to take responsibility to demonstrate that substances or products produced and placed on the market do not have any harmful effect on human or animal health or any unacceptable effects on the environment. Next to the legislation concerning the authorisation of pesticides, European regulations list the pesticide Maximum Residue Levels (MRLs) for different food products, but no such levels are set for adjuvants. Although adjuvants occur in large quantities in the environment only two products, nonylphenol and 4-nonylphenol, are listed as priority chemicals in the water framework directive [5]. This lack of regulation exists mainly because the applied adjuvants in a pesticide formulation are protected by industry are not disclosed to the public. Consequently, hardly any information on the toxicity, toxicological mode of action and environmental fate is available for authorities and the public. Furthermore, a lot of adjuvants are mixtures of different compounds and cause a lot of analytical challenges. Only very recently, US EPA considered requiring public disclosure of all ingredients of pesticide formulations [6], [7].

Most studies regarding adjuvants focus on the efficacy and only few research papers focus on toxicity and environmental fate. Nevertheless, there is an urgent need for information concerning the toxic mode of action, residue levels and the environmental fate of adjuvants for correct risk assessment and estimation of threshold levels [8]. Information on the toxic mode of action of compounds is important to develop a solid scientific basis for risk assessment [9], [10]. The use of appropriate alternative *in vitro* systems, can provide relevant information to facilitate regulatory decision-making. Moreover, the use of non-animal tests is promoted by the new EU crop protection regulation (1107/2009). The European OSIRIS project (Optimised strategies for risk assessment of industrial chemicals through integration of non-test and test information), proposes that a good way to improve the evaluation of chemicals may be by categorisation in modes of toxic action [11]. In this way, priorities for the evaluation of compounds can be set based on the toxic modes of action like for example the genotoxic potential of a compound. The *in vitro* assay used in this study is an example of such a test system. The multiple endpoint bacterial reporter assay is based on the induction of specific signalling pathways (oxidative stress, DNA damage, membrane damage and general cell lesions) that are universal in the living cell and hence the assay is able to combine the detection of toxic compounds and at the same time provide information on a number of universal mechanisms of toxicity [12].

In the present study we applied the bacterial multiple endpoint reporter assay to evaluate different adjuvants at the toxicity (growth inhibition) and toxic mode of action level. In a first step, bacterial growth inhibition (IC_50_, NOEC and LOEC) is quantified and compared between the different adjuvants.

Secondly, new information regarding different mechanisms of toxic action, i.e. DNA damage, oxidative stress, membrane damage and general cell lesions is obtained and these results are applied to categorise the adjuvants according to the mechanisms of toxic action. The toxicological results (acute toxicity and toxic mode of action) of this study are applied to select adjuvants that have a preference of use.

## Materials and Methods

### Selection of compounds

The different adjuvants were selected based on their high frequency of use in pesticides in Belgium (consumption data 2003). A broad selection was made containing compounds from the major adjuvant categories (Figure 1). To this selection of adjuvants, toxicological model compounds were added, i.e. mitomycin C (MytC) and methyl methane sulphonate (MMS) for DNA damage, hydrogen peroxide (H_2_O_2_) and paraquat (PQ) as model compounds for oxidative stress, and pentachlorophenol (PCP) and lindane (Li) for membrane damage and general cell lesions. The different solvents and model compounds were of analytical quality and obtained from Sigma (Sigma-Aldrich, Bornem, Belgium).

### Bacterial strains

All bacterial strains used, except *SfiA* are based on an *Escherichia coli* K-12 derivative SF1 containing the mutations *lac4169* deleting the entire *lac* operon, and *rpsL*. All the different *LacZ* fusions are present as single chromosomal inserts [13]. A selected list of strains from the publication by Orser et al. were used responding to different types of stress like DNA damage, oxidative stress, protein denaturation, membrane damage, osmotic stress, general cellular stress and heavy metal presence (Table 1). The *SfiA* strain is part of the SOS chromotest derived from *E. coli* GC4436 with a deletion in the *lac* operon carrying a sfiA:: lacZ fusion so that responses to DNA damaging agents can be measured [14].

**Table 1:** Stress gene promoters fused to the LacZ gene and their functional grouping (modified from Dardenne et al., 2007 and Orser et al., 1995).

Type of stress response	Promoter	Gene product/Function	Responsive to
Oxidative stress	KatG	Hydrogen peroxidase I	Oxidative stress
	Zwf	Glucose-6-phosphate dehydrogenase	Oxidative stress
	Soi28	Superoxide inducible gene	Superoxide radical generating agents
	Nfo	Endonuclease IV	Ss and dsDNA breaks, oxidative DNA damage
Membrane damage	MicF	Antisense RNA to 5′ OmpF	Membrane integrity, osmotic stress
	OsmY	Periplasmic Protein	Osmotic stress
General cell lesions	UspA	Universal stress protein	Growth arrest
	ClpB	Proteolytic activation of ClpP	Protein perturbation
Heavy metal stress	MerR	Regulation of the mercury resistance operon (mer)	Heavy metals
DNA Damage	Nfo	Endonuclease IV	Ss and ds DNA breaks, oxidative DNA damage
	RecA	General recombination and DNA repair	SOS response
	UmuDC	DNA repair	Radiation and/or chemically induced DNA damage
	Ada	Adaptive response to alkylation	DNA damage, mainly methyl adducts
	SfiA	Inhibitor of cell division	SOS response
	DinD	Unknown function within the DNA damage inducible response	DNA damage

### Toxicity evaluation of the selected adjuvants

The growth inhibition test was performed with the *E. coli ClpB* strain. The *ClpB* strain is a growth inhibition sensitive strain for a broad range of chemicals [12]. Pre-cultures were grown overnight at 37°C and 250 rpm in Luria Bertani (LB) broth medium (Sigma-Aldrich, Bornem, Belgium). Subsequently bacteria were exposed in 96-well plates for 90 minutes at 37°C and 200 rpm (detailed protocol described in [12]. Exposure experiments were carried out in 96-well plates in a linear ½ dilution series containing seven nominal concentrations (Table 2). Six replicates were performed for each exposure experiment and control (received only growth medium) and solvent control (growth medium and pure water) were included. Growth inhibition was calculated as the ratio of exposed versus non-exposed cell yield, expressed by the measured pre- and post-exposure optical density at 600 nm.

**Table 2:** Bacterial growth inhibition of adjuvants with used abbreviations throughout the study, concentration range tested (g/L), respective CAS number, IC50 values (concentration at which 50% of the bacteria stopped growing) with confidence intervals (CI), NOEC and LOEC (no and lowest observed effect concentration) at the growth inhibition level.

	abbreviation	concentration range (g/L)	CAS-number	IC50 (CI) g/L	LOEC g/L	NOEC g/L
**SURFACTANTS**						
Ethoxylated tallow alkyl amine	POEA	0.010–0.070	CAS 68478-96-6	0.019 (0.018–0.021)	0.010	<0.010
Ethoxylated fatty alcohol (AE7)	AE	0.00156–0.1	CAS 68002-97-1	0.039 (0.029–0.052)	0.013	0.006
Trisiloxaan ethoxy-propoxylate tenside	Tri EO-PO	0.0156–1	CAS 134180-76-0	0.082 (0.060–0.11)	0.031	0.016
Ethoxylated phosphate ester (isotridecanol)	Eo PE	0.078–5	CAS 9046-01-9	0.775 (0.69–0.86)	0.156	0.078
Ethoxylated fatty acid (isotridecanol)	Eo FA	0.312–20	CAS 9043-30-5	2.02 (1.67–2.45)	0.531	<0.531
Trisiloxaan ethoxylate tenside	Tri EO	0.023–1.5	CAS 27306-78-1	>1.5 (p)	0.468	0.234
Ethoxylated tristyrylphenol	Eo TP	0.14–9	CAS 99734-09-5	>0.63 (s)	>0.63	≥0.63
Ethoxylated nonylphenol	Eo NP	0.0078–0.5	CAS 9016-45-9	>0.5 (p)	0.015	0.008
**SOLVENTS**						
Isophorone	Is	0.562–36	CAS 78-59-1	3.98 (3.40–4.618)	0.563	<0.562
N-methyl-2-pyrrolidone	Pyr	0.662–42.4	CAS 872-50-4	11.84 (9.32–15.03)	0.66	<0.66
γ-butyrolactone	But	0.6–44	CAS 96-48-0	>44 (s)	>44	≥44
Dichloromethane	Di	0.0001–0.0015	CAS 75-09-2	>0.0015 (s)	>0.0015	≥0.0015
Isopropanol	Isp	1.2–100	CAS 67-63-0	>100 (s)	>100	≥100

If IC50 could not be calculated, the reason was mentioned (p = precipitation, s = solubility).

**Figure 2:**
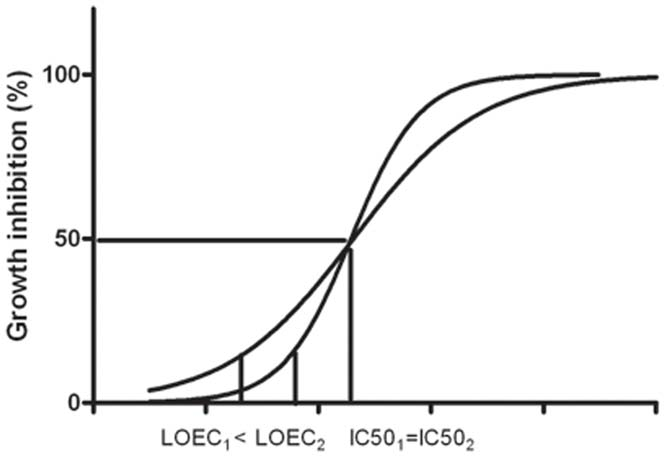
Description of differences in dose-respons curves, IC50 concentrations are equal but LOEC values differ due to differences in slope.

The standard to evaluate toxicity of a compound is based on the comparison of LC_50_, EC_50_ or IC_50_ values (concentration at which 50% of the test species die (L), are immobile (E) or stop growing (I)) obtained after exposure of the test species to a serial dilution of the selected compounds. This single value is not enough to characterise toxicity if the obtained dose response curves show differences in slopes, as compounds can be equitoxic based on IC_50_ values but the dose-response and hence slopes can be different (Figure 2). A supplementary value characterising toxicity at lower concentrations gives additional information, i.e. the NOEC and LOEC (no and lowest observed effect concentration).

For each compound, IC_50_ values were calculated using the logistic 4 parameter regression curve (GraphPad Prism). Lowest observed effect concentrations (LOEC) and NOEC at the level of growth inhibition were statistically derived using ANOVA and post hoc Dunett's test (p<0.05).

### Toxic mode of action evaluation of the selected adjuvants

The toxic mode of action of the different selected compounds was evaluated with a bacterial multiple endpoint reporter assay (Table 1). Concentrations for the toxic mode of action studies were based on the results from the growth inhibition experiments, i.e. highest test concentrations chosen were IC_20_ values. The bacterial reporter assay was performed as previously described [12], [15]. The assay was performed in triplicate in 96 well plates, column 2 till 11 received a uniform amount of the different overnight *Escherichia coli* cultures diluted in Luria Bertani (LB) medium, column one was used as a blank and only received LB. Optical density was measured at 600 nm to check uniformity. After 90 minutes of resuscitation (37°C and 200 rpm) the plates received the compound to be tested at different concentrations, optical density (600 nm) was measured before and after dosing. Columns 5 to 11 received an increasing concentration of the compound in a ½ serial dilution, columns 2 to 4 were negative controls. After 90 minutes of exposure (37°C and 200 rpm) optical density (600 nm) was measured again and the cells were lysed for β-galactosidase measurement. The reduction of ONPG (O-nitrophenyl- β-D-galactopyranoside) (colorless) to ONP (O-nitrophenol) (yellow) by β-galactosidase was measured spectrophotometrically at 420 nm and was used as a measure for activity of the promoters. Activity of the promoter was calculated taking into account the growth inhibition of the used strain. The results are presented as fold inductions at a given dose i, relative to the control values and were calculated through a set of formulas as given below [12]:
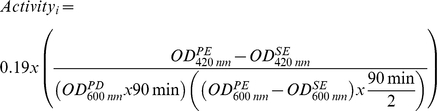
Formula 1 Activity at a given dose i. OD: optical density, PE: Post exposure, SE: start exposure ( = post dose), PD: pre-dose.
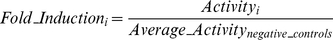
Formula 2 Fold Induction at a given dose i.

The presented fold inductions are the mean of three independent replicates. Fold inductions were considered significant when the following criteria were met: (a) presence of a concentration response relationship (R^2^>0.5, significant at p<0.05 for six degrees of freedom) and a positive slope different from 0 (p<0.05) in a linear model, (b) signal statistically significantly higher than the blank (Dunnett's test p<0.05) [16]. If fold inductions were not significant they were set to 1 to enhance readability of the data and to reduce noise.

### Toxicity and toxic mode of action classification

An initial ranking of the selected adjuvants was made based on the obtained toxicity and toxic mode of action data. Toxicity of compounds was characterised by IC_50_ and statistically derived NOEC and LOEC values. To characterise the toxic mode of action, the different stress responses were grouped into four major classes (Table 1), heavy metal response was left out. The promoter *MerR* was not considered for further analysis since it strongly and specifically reacts to specific heavy metal ions i.e. mercury and cadmium, and no such inductions were observed in the dataset.

In classical mortality tests 100% lethality can always be achieved if solubility of the test compound is not a limitation, however this is not the case at the gene expression level. The maximum induction level of a gene is not known and depends on the regulatory mechanism of the gene and the nature of the inducing compound, hence EC_x_ and toxic units have no direct biological meaning in this case. Consequently, a different approach was used to quantify the information at the gene expression level. If fold inductions were significant (criteria see above), the response for each gene was characterized by: 1) the fold induction scores (FIS) at the IC_20_ level, defined as the ratio of the measured FI to the reference compound FI (set to 100%) and 2) the LOEC at the gene expression level.

Principal component analysis (PCA) was performed using SIMCA-p v11.5 software, (Umetrics AB, Umea, Sweden) to assess similarities between cases. This multivariate approach allows the visualization of (combination of) mode(s) of action to which the adjuvants belong since the reference compounds were included in the dataset. The FIS dataset was used for PCA analysis, if inductions were not significant, FIS was set to 1, reference gene inductions of the model compounds were set to 100%.

## Results

### *Escherichia coli* growth inhibition (Table 2)

Lowest IC_50_ values are found for ethoxylated tallow alkyl amine (19 mg/L), ethoxylated fatty alcohol (39 mg/L) and trisiloxaan ethoxy-propoxylate tenside (82 mg/L) (Table 2). Due to solubility and precipitation problems, IC_50_ values cannot be calculated for 3 surfactants (trisiloxaan ethoxylate tenside, ethoxylated tristyrylphenol and ethoxylated nonylphenol) and 3 solvents (gamma-butyrolactone, dichloromethane and isopropanol). Nevertheless, LOEC and NOEC values could be calculated for ethoxylated nonylphenol (15 mg/L and 7.5 mg/L respectively) and trisiloxaan ethoxylate tenside (468 mg/L and 234 mg/L respectively). On the basis of the IC_50_ values one would conclude that ethoxylated nonylphenol is one of the non-toxic surfactants, but NOEC-LOEC calculations show that already at low concentrations growth inhibition (20%) is observed.

### Toxic mode of action

**Figure 3:**
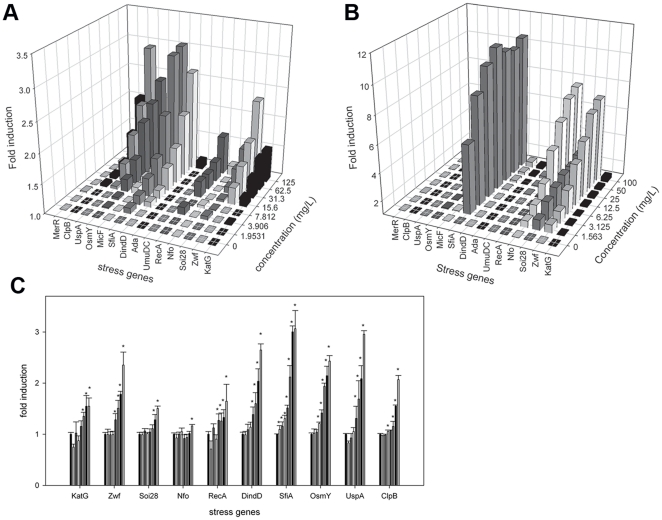
Bacterial dose response profile after exposure to an adjuvant (ethoxylated nonylphenol) and a reference compound (paraquat). Figure 3a) ethoxylated nonylphenol, and 3b) paraquat. The y-axis denotes the induction at any given dose, the x-axis shows the different stress genes and the z-axis shows the applied concentrations in a ½ serial dilution. All data are means of three replicates (n = 3), c) Detailed results for significantly induced genes after exposure to ethoxylated nonylphenol meeting the criteria as mentioned in Material and Methods, bars indicate standard error. *Significantly different from solvent control (one-way ANOVA, Dunett's test, p<0.05).

Next to toxicity data for the selected adjuvants more information regarding their toxic mode of action was obtained through a bacterial reporter assay with 14 different toxicologically relevant stress genes. The dose response profile after exposure to ethoxylated nonylphenol (Figure 3A) showed clear concentration responses for 10 stress genes, a detailed figure of the significantly induced genes with standard error is given in Figure 3C. The significantly induced genes belong to different toxic modes of action, oxidative damage (*KatG*, *Zwf*, *Soi28* and *Nfo*), DNA damage (*RecA*, *DinD* and *SfiA*), membrane damage (*OsmY*) and cellular stress (*ClpB* and *UspA*). The induced genes show a 3 fold induction at IC20 concentrations for *SfiA* and *UspA* and a 2.5 fold induction for *Zwf*, *DinD* and *OsmY*. Compared to the induction profile of ethoxylated nonylphenol the bacterial gene expression profile after exposure to the reference compound paraquat, induced a specific oxidative stress response (*Zwf*, *Soi28*, *Nfo* and *SfiA*) and the fold inductions are much higher i.e. up to 10-fold inductions (Figure 3B).

As mentioned above for gene expression data the maximum fold induction is not known, hence relative values are used, i.e. fold induction scores (FIS) (Table 3). These values can be compared since they represent gene expression at equitoxic concentrations. The individual fold inductions are given as supporting information (Table S1).To characterise the results of the dose response curves at lower concentrations, LOEC values are calculated (Table 4).

**Table 3:** Significantly induced effects at the gene expression level after exposure to the selected adjuvants.

	Oxidative damage				DNA damage					Membrane damage		General cell lesions	
	Kat G	Zwf	Soi 28	Nfo	Rec A	Umu DC	Ada	DinD	SfiA	Mic F	Osm Y	UspA	Clp B
**ADJUVANTS**													
POEA	128	68	281	300	58	106	20	54	-	240	275	101	75
AE	213	90	57	15	56	14	52	-	253	52	319	231	441
Tri EO-PO	-	-	121	40	-	-	15	-	-	-	-	-	-
Eo PE	55	19	-	-	-	-	21	-	81	-	47	-	161
Eo FA	-	-	67	34	48	-	19	58	-	169	149	-	56
Tri EO	-	47	78	-	57	-	14	-	136	-	-	-	-
EO TP	-	-	-	-	-	-	-	54	-	-	-	-	55
EO NP	71	33	44	15	35	-	29	-	146	-	72	159	89
Is	71	-	-	-	-	-	-	-	-	-	-	-	-
Pyr	166	21	-	16	-	13	11	18	-	-	-	83	-
But	-	22	37	16	30	11	12	16	-	-	-	69	56
Di	-	17	-	-	25	-	-	-	-	-	-	-	-
Isp	-	-	-	-	-	-	-	15	-	-	-	-	-

Results are expressed as fold induction scores (FIS) (%), calculated as the ratio of the measured fold induction (FI) at IC20 level to the reference compound FI at IC20 level.– not significantly induced.

**Table 4:** Statistically derived no observed effect concentrations (NOEC) (g/L) at the level of gene expression for significantly induced genes (ANOVA, post hoc Dunett's test p<0.05).

	Oxidative damage				DNA damage					Membrane damage		General cell lesions	
	Kat G	Zwf	Soi 28	Nfo	Rec A	Umu DC	Ada	DinD	SfiA	Mic F	Osm Y	UspA	Clp B
**ADJUVANTS**													
POEA	8,0E−05	8,0E−05	8,0E−05	4,0E−05	2,0E−05	2,0E−05	1,6E−04	-	2,0E−05	8,0E−05	2,0E−05	8,0E−05	8,0E−05
AE	3,0E−02	1,6E−03	1,3E−02	2,5E−02	3,1E−03	2,5E−02	-	3,1E−03	2,5E−02	2,5E−02	1,6E−03	1,6E−03	1,6E−03
Tri EO-PO	-	-	3,0E−03	2,0E−02	-	-	-	-	1,0E−03	-	-	-	-
Eo PE	6,3E−01	1,3E+00	-	-	-	-	-	1,6E−01	1,6E−01	-	1,6E−01	-	1,6E−01
Eo FA	-	-	1,3E+00	1,3E+00	3,1E−01	-	1,3E+00	-	1,3E+00	6,3E−01	1,6E−01	-	3,1E−01
Tri EO	-	2,5E−03	4,0E−02	-	6,0E−04	-	-	2,0E−02	1,3E−03	-	-	-	-
EO TP	-	-	-	-	-	-	5,6E−01	-	-	-	-	-	4,5E+00
EO NP	1,6E−02	1,6E−02	6,3E−02	1,3E−01	1,6E−02	-	-	1,6E−02	2,0E−03	-	8,0E−03	1,6E−02	1,6E−02
Is	1,3E+00	-	-	-	-	-	-	-	-	-	-	-	-
Pyr	6,3E−01	2,5E+00	-	1,6E−01	5,0E+00	5,0E+00	1,3E+00	-	2,5E+00	-	-	5,0E+00	-
But	-	5,0E+00	2,5E+00	2,5E+00	5,0E+00	1,0E+01	1,0E+01	-	5,0E+00	-	-	1,0E+01	5,0E+00
Di	-	1,5E−03	-	-	1,5E−03	-	-	-	-	-	-	-	-
Isp	-	-	-	-	-	-	5,0E+01	-	-	-	-	-	-
**REFERENCE COMPOUNDS**													
MytC	6,3E−04	-	-	-	8,0E−05	4,0E−05	-	-	1,3E−03	-	-	-	-
MMS	-	-	-	-	5,0E−02	3,1E−03	5,0E−02	-	-	-	-	-	1,0E−01
PQ	1,3E−02	1,6E−03	1,6E−03	1,6E−03	-	2,5E−02	5,0E−02	1,3E−02	1,6E−03	-	2,5E−02	5,0E−02	-
H2O2	1,0E−04	2,1E−03	-	-	2,1E−03	2,1E−03	-	-	-	-	-	-	-
PCP	-	1,5E−03	-	-	-	-	1,5E−03	-	1,5E−03	2,0E−05	-	-	9,0E−05
Li	1,3E−03	2,5E−03	-	-	2,5E−03	-	-	2,5E−03	-	-	2,5E−03	2,5E−03	-

Two important groups of adjuvants are evaluated in this study, solvents and non-ionic surfactants, the results show that in general much lower inductions are found for solvents than for surfactants. The observed LOEC values for the tested solvents are much higher (g/L range) than for surfactants (mg/L range), illustrating that the effect concentrations are much higher for solvents than for surfactants (Table 4).

All tested surfactants, except EO TP, exceeded the 100% level for one or more genes indicating that they provoke higher inductions than the reference compounds. For the selected solvents only Pyr exceeded the 100% level for *KatG*. It is clear from the FIS at IC20 values that POEA and EA provoke far more stress responses than the other surfactants and solvents. The related LOEC values illustrate that the effects at the gene expression level appear at low concentrations, ranging from 20–80 µg/L for POEA and from 1.6–25 mg/L for EA (Table 4).

The markers for membrane damage are not induced after exposure to the selected solvents. The SOS response related genes *RecA*, *UmuDC* and *SfiA* are induced after exposure to Pyr and But, mild SOS responses for EO FA, tri EO and EO NP and severe SOS responses, *RecA* and *UmuDC* inductions, after exposure to POEA and AE (Table 3).

### Categorization into toxic mode of action

**Figure 4:**
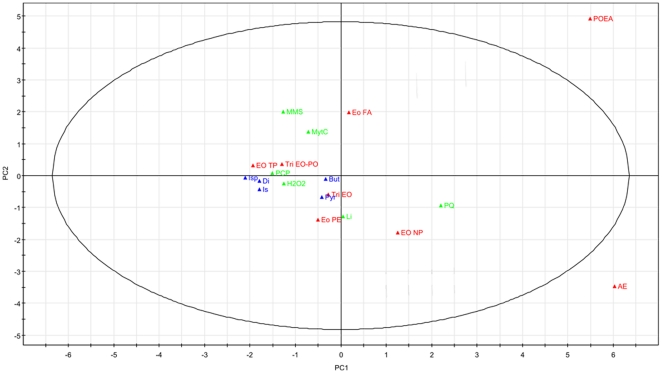
Principal component analysis of FIS (fold induction score) dataset. The first two components (PC1 and PC2) are shown. Individual points represent the gene expression pattern. This plot shows the possible presence of outliers, groups, similarities and other patterns in the data. Observations situated outside the ellipse are outliers. Blue dots: solvents, red dots: surfactants, green dots: reference compounds.

Compared to the reference compounds which show a principal mode of action, i.e. the reason why they are considered model compounds, the adjuvants show “mixed” toxic modes of action (Table 3, 4 and Figure 4). The toxic mode of action of POEA and EA is complex with inductions of all classes of stress genes making it impossible to assign one or more principal mechanisms of action to those compounds.

Principal component analysis on the FIS dataset illustrates that POEA and AE are grouped separately from all the other compounds and the software labeled them as possible outliers (Figure 4). In the obtained model (R^2^ = 0.66 ) the first principal component (PC1) explains the majority of the variance (41%) and describes the difference in the SoxRS mediated oxidative stress response on one hand and the OxyR oxidative stress response and membrane damage response on the other hand. The second component (24%) separates DNA damage markers from oxidative damage and membrane damage markers. The data points that are grouped together are isoforon, isopropanol dichloromethane, pentachlorophenol, hydrogen peroxide and ethoxylated tristyrylphenol, for these compounds the FIS profiles show low inductions. Ethoxylated fatty acid is grouped together with DNA damage inducers MMS and MytC, mostly because the *Ada* response is induced, yet the FIS show main inductions for membrane damage related genes.

## Discussion

### Toxicity and toxic mode of action of adjuvants

Adjuvants comprise of three major groups: surfactants, solvents and synergists and are often referred to as “inert ingredients”. A consumer survey performed by US EPA learned that many consumers are mislead by the term “inert ingredient”, believing it to mean harmless [17]. This certainly is not the case and in fact they can be toxic to humans, may have biological activity of its own [18], [19]. Nevertheless, up till recently adjuvants were not taken into account for the risk evaluation of pesticides. The upcoming new EU regulation concerning the placing of plant protection products on the market (EC1107/2009) includes for the first time the demand for information on the possible negative effects of not only the active ingredients but also the used adjuvants. This new regulation requires basic toxicological information that is used to decide on the use, ban or preferential use of available adjuvants [8].

This study provides information on the toxicity and toxic mode of action of the selected compounds. The ranking of the adjuvants based on their toxicity (growth inhibition) showed that the surfactants are far more toxic than the selected solvents in the assay. Ethoxylated tallow alkyl amine is the most toxic compound tested. High toxicity after exposure to ethoxylated tallow alkyl amine was already reported for several species e.g. tadpoles and green algae [20]–[23]. Within the group of surfactants toxicity varies by three orders of a magnitude, with ethoxylated fatty acid (isotridecanol) and trisiloxaan ethoxylate tenside as the least toxic compounds. The toxicity results illustrate the importance of reporting toxicity in different ways (here IC_50_ and NOEC-LOEC) to characterise the toxicity of a compound. If only IC_50_ values are determined EO NP would be regarded as a non-toxic compound while growth inhibition already occurs at low concentrations. For several compounds IC_50_ and LOEC values could not be calculated due to limited water solubility. We preferred not to use other solvents than water since in realistic conditions (sprays and tank-mixes) water is used as a diluent or solvent.

Organosilicone surfactants, a fairly new class of non-ionic wetting agents, do not act like classical surfactants through the membranes but they provide a faster penetration of the pesticide in the plant through a specific mode of action i.e. by facilitating stomatal infiltration of solutions [24]. They are considered as promising compounds since improved spreading of the active ingredient can lead to a reduction of the latter in formulations. Two organosilicone surfactants were tested in this study, i.e. trisiloxane ethoxylate tenside (tri EO) and trisiloxane ethoxy-propoxylate tenside (tri EO-PO). Both compounds increase the uptake and efficacy of pesticides in a similar way [25], though this study demonstrates that they differ by one order of magnitude at the toxicity level. Stark and Walthall (2003) investigated the acute toxicity of several agricultural adjuvants, including organosilicone surfactants, with *Daphnia pulex*. They found different LC_50_ values for different organosilicone surfactants: Silwet L-77® 3 mg/L and Kinetic® 111 mg/L. The results from our study at the gene expression level confirm that the main mode of action of the tested organosilicone surfactants is not through membrane damage (*MicF*, *OsmY* and *ClpB*) since these genes are not significantly induced. The toxic mode of action of organosilicone surfactants is mainly oxidative damage through part of the SoxRS pathway (*Zwf* and *Soi28*). Both compounds are not grouped together with paraquat, the model compound for SoxRS mediated oxidative damage, in the PCA analysis the reason for this is that not the whole SoxRS pathway is induced as can be observed from the FI(S) dataset.

In a mode of action and QSAR (quantitative structure activity relationships) context, non-ionic surfactants are described as compounds that provoke toxicity through non-specific mechanisms, the toxic potency of these compounds correlates well with their hydrophobicity. Such a mode of action is defined as narcosis, one of the four mode of action categories (narcotics, non-polar narcotics, reactive chemicals and specifically acting reactive chemicals) in the Verhaar classification scheme (Verhaar et al., 2000). Exposure to narcotics typically results in disruption of the biological membrane integrity [26], [27]. Several of the non-ionic surfactants included in this study induced membrane damage (*MicF* and *OsmY*) and general cell lesions (*UspA* and *ClpB*). Narcosis was already described for several of the adjuvants tested, dichlormethane, ethoxylated nonylphenol, ethoxylated alcohol [20], [28]. The results in our study confirmed these results for EA and EO NP and also revealed membrane damage after exposure to POEA, EO PE and gamma-butyrolactone. In our study, no membrane damage is found after exposure to dichloromethane, but the test concentrations were low due to the limited solubility.

Membrane damage and general cell lesions were not the only pathways affected after exposure to these compounds, DNA damage and oxidative stress are induced as well. The induced DNA damage markers are part of the SOS response, a well described repair mechanism in bacteria [29]. Valuable markers for the SOS response are *RecA*, *UmuDC* and *SfiA*, they can be considered as indicators for potential genotoxic compounds like the model compound methylmethane sulphonate (MMS) [12]–[14]. *SfiA*, is also part of the validated SOS chromotest [14]. The observed DNA damage (both FIS and NOEC) demonstrated that in the reporter assay the SOS response pathway is induced as described in literature, mild SOS response only *RecA* induction and severe SOS response both *RecA* and *UmuDC* inductions [29]. Previous studies already pointed out that the induction of *SfiA* could be related to oxidative DNA damage [12]. This is also the case in our study since together with the high induction of the oxidative damage markers the induction of *SfiA* was observed.

Several of the surfactants (POEA, AE, tri-EO, EO FA and EO NP) and one solvent (gamma-butyrolactone) that were tested showed significant inductions for the SOS response pathway. The FIS showed for several compounds inductions of up to 50% of the MMS signal for *RecA*, indicating that POEA, AE, EO FA and tri EO are half as potent as MMS to induce *RecA*. These results were observed at mg/L range for POEA, EA and tri EO and in g/L range for EO FA. Environmental concentrations of the selected compounds are not routinely monitored so little data are available, Belanger and colleagues found concentrations of AE (sum of all) in European effluents of 6.8 µg/L, far below the LOEC at the gene expression level, nevertheless further research on the potential genotoxic effects of these compounds is needed as there are no threshold levels for genotoxic compounds [30].

The most recent US-EPA classification of adjuvants lists gamma butyrolactone as harmless and the usage in pesticides is unlimited, though the report lists genotoxic effects at high concentrations [31]. The concentrations that were tested in this study are very high and unlikely to occur in the environment or food chain. Information on possible genotoxic potential of the other tested compounds is not found in literature.

Ethoxylated fatty alcohol is considered as an alternative for the endocrine disruptor ethoxylated nonylphenol which is banned in Europe. Nevertheless, toxicity results in this study show that NOEC-LOEC values are comparable [20]. At the gene expression level, both compounds induce several stress genes, the LOEC at the gene expression level is even lower for ethoxylated fatty alcohol than for ethoxylated nonylphenol. Based on the results from this study other surfactants seem more appropriate to replace ethoxylated nonylphenol i.e. organosilicone surfactants or ethoxylated tristyryl phenol. The results show a first ranking based on toxicity and toxic mode of action of adjuvants, but additional information concerning other relevant endpoints like endocrine disruption potential is needed.

### Future perspectives of toxic mode of action studies for ranking of chemicals

Information on environmental concentrations of surfactants is very scarce, moreover for most adjuvants the persistence, bioaccumulation rates and effects in aquatic and terrestrial systems are not known. However, this information is necessary for correct risk assessment. The results from this study provide important information on the effects (toxicity and toxic mode of action) of environmentally important adjuvants. Nevertheless, this study also illustrates that most compounds do not trigger the induction of one specific mode of action, but a combination of several pathways. The interpretation of such results requires expert judgment since the categorization into toxic modes of action is difficult with mixed modes of action, e.g. a compound can be genotoxic and cause membrane damage. In this case the genotoxic properties are more important for the environment and human population, but other combinations of modes of action are possible as well, a compound can provoke narcosis (membrane damage) and have endocrine disrupting potential. Powerful clustering and multivariate statistics are necessary to interpret such complex information and these are important challenges for the use of mechanistic information and categorization into toxic modes of action.

### Conclusions

In this study a bacterial multiple endpoint reporter assay with universally stress related endpoints was used to obtain more information on the toxicity and toxic mode of action of several agricultural adjuvants. The results show that the selected solvents are less toxic than the surfactants, suggesting that solvents may have a preference of use, but further research on more compounds is needed to confirm this observation. The gene expression profiles of the selected surfactants reveal that a phenol (ethoxylated tristyrylphenol) and an organosilicone surfactant (ethoxylated trisiloxane) show little or no inductions at EC_20_ concentrations, making them preferred surfactants for use in different applications. The organosilicone surfactant is a fairly new compound that looks very promising, with little or no toxicity and good adjuvant properties.

However, this study also illustrates severe effects at the level of DNA damage with the induction of the bacterial SOS response indicating possible genotoxicity for several of the surfactants (POEA, AE, tri-EO, EO FA and EO NP) and one solvent (gamma-butyrolactone). For several compounds the FIS show inductions of up to 50% of the MMS signal for *RecA*, indicating that POEA, AE, EO FA and tri EO are half as potent as MMS to induce *RecA*.

Using the information at the gene expression level, we attempted to assign a principal mode of action to the selected adjuvants using multivariate statistics. The principal component analysis revealed that most compounds show a mixed mode of action and AE and POEA show such high inductions for several stress genes that they are allocated as outliers. The technique that was applied shows promising perspectives for the classification of compounds and classification will improve as the dataset expands.

## Supporting Information

Table S1 Significant gene inductions after exposure to the selected adjuvants and reference compounds. Results are expressed as fold induction (FI) at IC20 level, non induced genes are set to 1.
(DOC)
