# Supplementary material for: Toxicity Ranking and Toxic Mode of Action Evaluation of Commonly Used Agricultural Adjuvants on the Basis of Bacterial Gene Expression Profiles
Source: PLoS One. 2011 Nov 18;6(11):e24139. doi: 10.1371/journal.pone.0024139 (PMC3220671; doi:10.1371/journal.pone.0024139)
Supplement: Table S1 — Significant gene inductions after exposure to the selected adjuvants and reference compounds. Results are expressed as fold induction (FI) at IC20 level, non induced genes are set to 1. (DOC) [file pone.0024139.s001.doc]

|  | **Oxidative damage** | | | | **DNA damage** | | | | | **Membrane damage** | | **General cell lesions** | |
| --- | --- | --- | --- | --- | --- | --- | --- | --- | --- | --- | --- | --- | --- |
|  | Kat G | Zwf | Soi 28 | Nfo | Rec A | Umu DC | Ada | DinD | SfiA | Mic F | Osm Y | UspA | Clp B |
| **ADJUVANTS** | | | | | | | | | | | | | |
| POEA | 2,81 | 4,8 | 9,59 | 23,16 | 2,77 | 11,26 | 4,68 | 0 | 2,13 | 5,87 | 9,3 | 1,86 | 1,73 |
| AE | 4,66 | 6,35 | 1,94 | 1,18 | 2,66 | 1,47 | 1 | 4,61 | 5,5 | 1,27 | 10,77 | 4,27 | 10,23 |
| Tri EO-PO | 1 | 1 | 4,13 | 3,07 | 1 | 1 | 1 | 1 | 1,62 | 1 | 1 | 1 | 1 |
| Eo PE | 1,21 | 1,32 | 1 | 1 | 1 | 1 | 1 | 1,48 | 2,26 | 1 | 1,58 | 1 | 3,73 |
| Eo FA | 1 | 1 | 2,27 | 2,64 | 2,29 | 1 | 4,95 | 1 | 2,05 | 4,15 | 5,04 | 1 | 1,29 |
| Tri EO | 1 | 3,32 | 2,66 | 1 | 2,69 | 1 | 1 | 2,47 | 1,51 | 1 | 1 | 1 | 1 |
| EO TP | 1 | 1 | 1 | 1 | 1 | 1 | 4,62 | 1 | 1 | 1 | 1 | 1 | 1,27 |
| EO NP | 1,55 | 2,35 | 1,5 | 1,16 | 1,65 | 1 | 1 | 2,65 | 3,06 | 1 | 2,43 | 2,95 | 2,07 |
| Is | 1,55 | 1 | 1 | 1 | 1 | 1 | 1 | 1 | 1 | 1 | 1 | 1 | 1 |
| Pyr | 3,64 | 1,49 | 1 | 1,23 | 1,3 | 1,33 | 1,58 | 1 | 1,19 | 1 | 1 | 1,54 | 1 |
| But | 1 | 1,52 | 1,27 | 1,27 | 1,43 | 1,19 | 1,39 | 1 | 1,28 | 1 | 1 | 1,28 | 1,3 |
| Di | 1 | 1,19 | 1 | 1 | 1,2 | 1 | 1 | 1 | 1 | 1 | 1 | 1 | 1 |
| Isp | 1 | 1 | 1 | 1 | 1 | 1 | 1,26 | 1 | 1 | 1 | 1 | 1 | 1 |
| **REFERENCE COMPOUNDS** | | | | | | | | | | | | | |
| MytC | 1,17 | 1 | 1 | 1 | 4,76 | 10,62 | 1 | 1 | 1,12 | 1 | 1 | 1 | 1 |
| MMS | 1 | 1 | 1 | 1 | 2,76 | 6,44 | 8,6 | 1 | 1 | 1 | 1 | 1 | 1,29 |
| PQ | 1,36 | 7,05 | 3,41 | 7,72 | 1 | 1,24 | 1,23 | 1,4 | 10,59 | 1 | 1,65 | 1,33 | 1 |
| H2O2 | 2,19 | 1,2 | 1 | 1 | 1,17 | 1,66 | 1 | 1 | 1 | 1 | 1 | 1 | 1 |
| PCP | 1 | 1 | 1 | 1 | 1 | 1 | 1 | 1 | 1 | 2,45 | 1 | 1 | 2,32 |
| Li | 1,84 | 2,04 | 1 | 1 | 1,14 | 1 | 1 | 1,82 | 1 | 1 | 3,38 | 1,85 | 1 |
